# Myofascial Release for Chronic Low Back Pain: A Systematic Review and Meta-Analysis

**DOI:** 10.3389/fmed.2021.697986

**Published:** 2021-07-28

**Authors:** Zugui Wu, Yi Wang, Xiangling Ye, Zehua Chen, Rui Zhou, Zixuan Ye, Jinyou Huang, Yue Zhu, Guocai Chen, Xuemeng Xu

**Affiliations:** ^1^The Fifth Clinical Medical College, Guangzhou University of Chinese Medicine, Guangzhou, China; ^2^The Second Clinical Medical College, Guangzhou University of Chinese Medicine, Guangzhou, China; ^3^Guangdong Second Traditional Chinese Medicine Hospital, Guangzhou, China; ^4^Baishui Health Center, Qujing, China; ^5^Foshan Hospital of Traditional Chinese Medicine, Guangzhou University of Chinese Medicine, Guangzhou, China

**Keywords:** myofascial release, complementary therapy, chronic low back pain, meta-analysis, review

## Abstract

**Background:** Chronic low back pain (CLBP) is one of the most common musculoskeletal diseases in the elderly, which has a severe impact on the health of the elderly. However, CLBP treatment is very challenging, and more effective treatment methods are needed. Myofascial release may be an effective therapy for the management of chronic musculoskeletal pain. It is widely used clinically to treat CLBP, but its clinical efficacy is still controversial.

**Objective:** This study aims to systematically evaluate the effectiveness of myofascial release for patients with CLBP.

**Methods:** We selected PubMed, Cochrane Library, EMBASE database, and Web of Science database articles published until April 5, 2021. Randomized controlled trials (RCTs) of myofascial release for CLBP were included. Outcome measures included pain, physical function, quality of life, balance function, pain pressure-threshold, trunk mobility, and mental health. For each outcome, Standardized mean differences (SMD) or mean differences (MD) and 95% confidence intervals (CIs) were calculated.

**Results:** Eight RCTs (*n* = 375) were included based on inclusion and exclusion criteria. The meta-analysis showed that the overall efficacy of myofascial release for CLBP was significant, including two aspects: pain [SMD = −0.37, 95% CI (−0.67, −0.08), *I*^2^ = 46%, *P* = 0.01] and physical function [SMD = −0.43, 95% CI (−0.75, −0.12), *I*^2^ = 44%, *P* = 0.007]. However, myofascial release did not significantly improve quality of life [SMD = 0.13, 95% CI (−0.38, 0.64), *I*^2^ = 53%, *P* = 0.62], balance function [SMD = 0.58, 95% CI (−0.49, 1.64), *I*^2^ = 82%, *P* = 0.29], pain pressure-threshold [SMD = 0.03,95% CI (−0.75, 0.69), *I*^2^ = 73%, *P* = 0.93], trunk mobility [SMD = 1.02, 95% CI (−0.09, 2.13), *I*^2^ = 92%, *P* = 0.07] and mental health [SMD = −0.06, 95% CI (−0.83, 0.71), *I*^2^ = 73%, *P* = 0.88].

**Conclusions:** In this study, we systematically reviewed and quantified the efficacy of myofascial release in treating CLBP. The meta-analysis results showed that myofascial release significantly improved pain and physical function in patients with CLBP but had no significant effects on balance function, pain pressure-threshold, trunk mobility, mental health, and quality of life. However, due to the low quality and a small number of included literature, more and more rigorously designed RCTs should be included in the future to verify these conclusions.

## Introduction

Chronic low back pain (CLBP) is one of the most common musculoskeletal diseases in the elderly, ranking third among various musculoskeletal diseases (1, 2). The underlying pathological causes of CLBP are still not clear. Some studies suggest that it is related to various factors, such as age, health status, psychological factors, occupation, etc. (3, 4). Due to the high incidence and recurrence rate of CLBP, it has caused a substantial social and economic burden on the patient, family, and society (5, 6). Therefore, it is essential to find an effective treatment for CLBP. The treatment of CLBP is very challenging. There are many ways to treat this disease in the clinical environment, such as surgery, medication, physical therapy, and exercise (7, 8). Non-steroidal anti-inflammatory drugs (NSAIDs) are one of the effective drug therapies. However, long-term use of these drugs can cause many adverse effects, such as gastrointestinal reactions and cardiovascular events (9, 10). Furthermore, surgical therapy often brings sequelae, such as postoperative CLBP and surgical failure (11, 12), so many refuse surgical therapy. Therefore, many doctors and patients are often looking for more effective ways to treat CLBP.

In recent years, there have been many explorations in treating CLBP by manipulation (13–16). As a manipulation method, myofascial release is a possible management method for chronic musculoskeletal pain (17) and has been widely used in clinical practice for CLBP (18). Previous studies have found that the psoas muscle fascia may be related to CLBP (19, 20). Myofascial release combined with other therapies can effectively reduce the pain and disability of patients with CLBP (21, 22). At the same time, other studies have shown that myofascial release affects the flexibility of patients with CLBP (23) and improves trunk mobility and balance function (16). However, some studies have shown that myofascial release does not improve the pain and disability of patients with CLBP (13) and does not affect the flexibility of the lower limbs, the balance of the body, and the quality of life of patients (23, 24).

Although the myofascial release is widely used to treat CLBP, its clinical efficacy is still controversial (25). In this case, systematic reviews and meta-analyses have not been performed. In recent years, many RCTs on myofascial release in the treatment of CLBP have been published. Therefore, it is necessary to conduct systematic reviews and Meta-analysis to evaluate its efficacy. This meta-analysis aimed to evaluate and analyze the efficacy of myofascial release in the treatment of CLBP. Several variables were compared, including pain, physical function, quality of life, balance function, pain pressure-threshold, trunk mobility, and mental health.

## Methods

This study was conducted in accordance with the PRISMA guidelines and the recommendations of the Cochrane Collaboration (26). All analyses were based on published data in previous studies, so ethical approval was not required. Systematic Review Registration: http://www.crd.york.ac.uk/prospero, identifier: CRD42021250618.

### Selection Criteria

#### Studies Types

This study included only randomized controlled trials (RCTs). Non-RCTs, observational studies, and systematic reviews were all excluded. The language of all included literature was restricted to English.

#### Patients

The study included patients with CLBP (more than three months). There were no restrictions on the age, gender, comorbidities, and diagnostic criteria used in patients with CLBP.

#### Interventions

RCTs with myofascial release as the main intervention were included. There are no restrictions on the specific way of myofascial release, the frequency of intervention, and the length of intervention time. When combined interventions were used in the study, all participants in the myofascial release group and control group received the same combined interventions before they were considered eligible.

#### Specific Comparisons

We searched for RCTs that included one of the following group comparisons.

1. Myofascial release vs. Sham myofascial release.
2. Myofascial release vs. Exercises.
3. Myofascial release + exercises vs. Exercises.
4. Myofascial release + spinal manipulation vs. Spinal manipulation.
5. Myofascial release + physiotherapy program vs. Physiotherapy program.

#### Outcomes

For inclusion in this review, RCTs had to assess at least one major outcome or minor outcome:

The major outcomes included:

1. Pain, as measured, used the visual analog scale (VAS) or McGill Pain Questionnaire (MPQ).
2. Physical function, as measured, used Quebec Back Pain Disability Scale (QBPDS), Roland Morris Questionnaire (RMQ), or Oswestry Disability Index (ODI).
3. Quality of life, as measured, used EuroQol-5-Dimensions-3-levels (EQ3D5L), the MOS 36-item Short-Form Health Survey (SF-36), or World Health Organization Quality of Life Instrument-Older Adults Module (WHOQOL-OLD).
4. Balance Function, as measured, used Y-Balance Test (YBT) or Functional Reach Test (FRT).
5. Pain pressure-threshold.

The minor outcome included:

1. Trunk mobility (Sagittal plane mobility and Coronal plane mobility).
2. Mental health,as measured used the Fear-Avoidance Beliefs Questionnaire (FABQ) or Tampa Scale of Kinesiophobia (TSK).

#### Search Strategy

We searched Medline, EMBASE, Cochrane library, and Web of Science databases until April 5, 2021. Search terms such as the following were used: “Chronic low back pain,” ”Low back pain,” “Non-specific low back pain,” “Myofascial release,” “Randomized Controlled Trial,” “Clinical Trial,” “Randomly,” “Randomized,” “Randomization,” “Controlled.” After the search was completed, four researchers conducted a preliminary screening by reading the title and abstract and then performed a strict screening after reading the full text. Finally, the included literature was determined according to the inclusion and exclusion criteria. Controversies in the literature screening process were discussed with the fifth researcher and reached a consensus. The detailed search strategy was in the Supplementary Appendix.

### Data Extraction and Quality Assessment

Four reviewers independently extracted study data from eligible studies, including patient characteristics (age and gender), study characteristics (study design, publication year, country, sample size, number of dropouts, length and frequency of intervention, and duration), and study results. Controversies in the data extraction process were discussed with the fifth researcher and reached a consensus.

### Assessment of Risk of Bias in Included Studies

Two researchers evaluated the quality of the included literature and the risk of bias. The evaluation was based on the Cochrane Handbook 5.1.0 version. The literature was evaluated from the following seven aspects: Selection bias (random sequence generation, allocation concealment), Performance bias (blinding of participants and personnel), Detection bias (blinding of outcome assessment), Attrition bias (incomplete outcome data), Reporting bias (selective reporting), Other bias (27). The disputes in the evaluation process were discussed with the third researcher and reached a consensus.

### Rating Quality of Evidence

We used the Grading of Recommendations Assessment, Development and Evaluation (GRADE) Tool to evaluate the quality of evidence for myofascial release for CLBP. According to GRADE guidelines, each outcome was evaluated. The evaluation level is divided into high, moderate, low, and very low.

### Statistical Analysis

We used Review Manager 5.3 software (Cochrane Collaboration, Oxford, UK) to perform statistical analysis on the extracted data and used a forest plot to display the results. The standard mean differences (SMDs) and 95% confidence intervals (CIs) were calculated by random-effects models or fixed-effects models. The heterogeneity test uses *I*^2^ and chi-square statistics for analysis. When *I*^2^ < 50%, it indicates that there is no significant statistical difference in heterogeneity, and a fixed-effect model was used for statistical analysis. When *I*^2^ ≥ 50% indicates a significant statistical difference in heterogeneity, a random-effects model was used for statistical analysis. Funnel plots were used to assess publication bias for included studies.

## Results

### Study Selection

**Figure 1:**
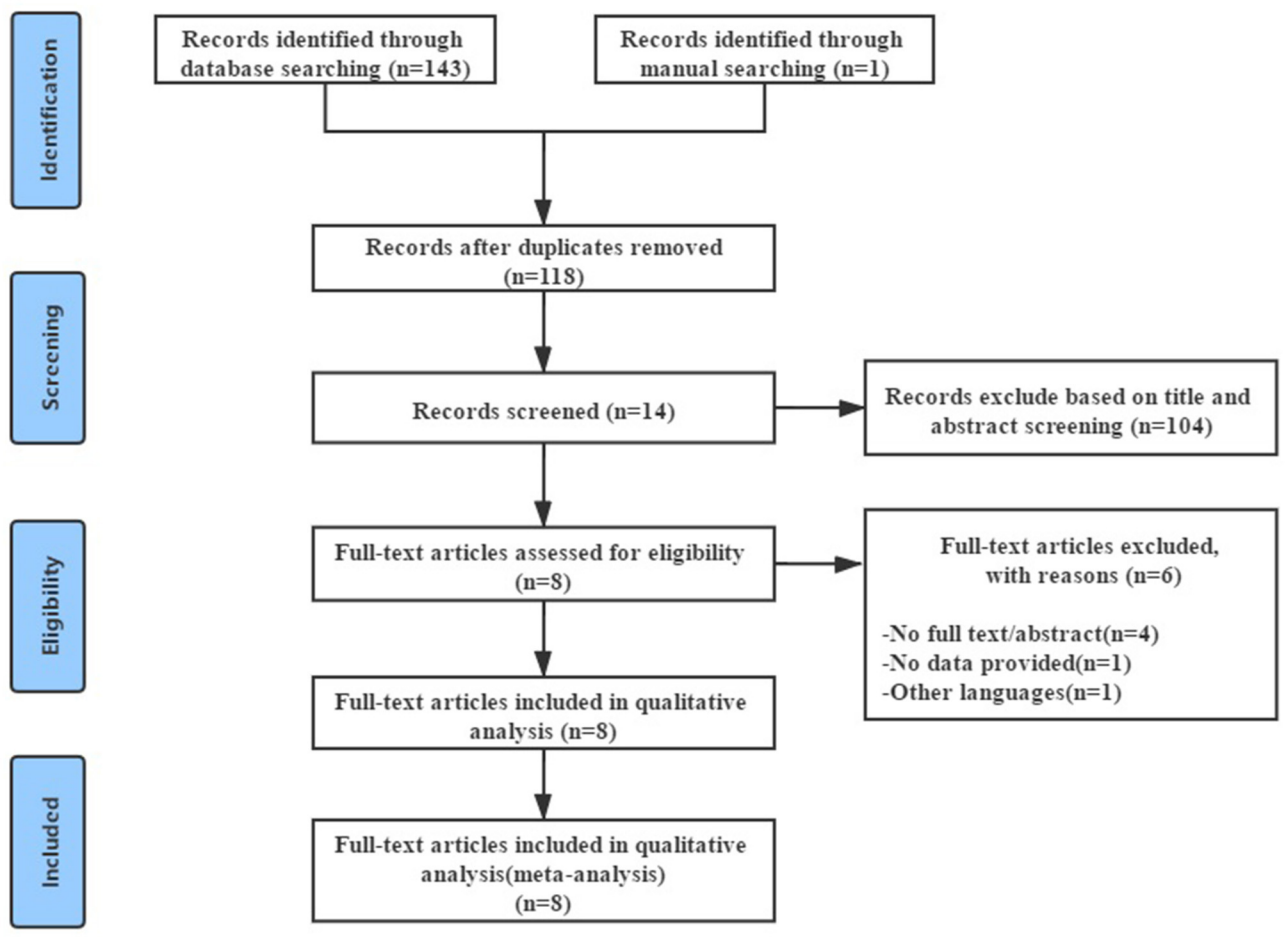
Flowchart of meta-analysis search and selection process.

Figure 1 showed the process of literature screening. By searching four English electronic databases, 144 relevant studies were selected, 26 duplicated studies were excluded after double-checking, 104 studies were excluded after reading the title and abstract, and the remaining 14 studies required further reading of the full text. Of the remaining fourteen studies, four were abstracts, one had no available data, and one was not published in English, leaving eight RCTs. Further reading of these eight RCTs confirmed that they met the inclusion criteria. Eight RCTs that met the inclusion criteria were included for meta-analysis, involving 375 patients with CLBP (13, 16, 18, 22–24, 28, 29).

### Study Characteristics

#### Overview of Included Studies

Table 1 summarized the characteristics of these eight RCTs, which were published between 2014 and 2020. The eight RCTs included involved six countries. Turkey (24), Brazil (13), India (22), and Italy (29) conducted one study, respectively, and Spain (18, 28) and Korea (16, 23) conducted two studies, respectively. In eight RCTs, 375 patients with CLBP were analyzed, 187 in the myofascial release group and 188 in the control group. Of the 375 patients with CLBP, 212 were female, and 163 were male. Mean age ranged from 34.2 (9.30) to 70.4 (3.20), sample size ranged from 24 to 74, and sample loss ranged from 0 to 8. No specific diagnostic criteria were reported in any of the studies.

**Table 1:** Study characteristics.

**References**	**Country**	**Study design**	**Mean age (SD), years**		**Sample size**		**Male/female**		**Drop out**	
			**MG**	**CG**	**MG**	**CG**	**MG**	**CG**	**MG**	**CG**
Ajimsha et al. (22)	India	RCT	35.80 (8.40)	34.20 (9.30)	38	36	9/29	8/28	2	4
Arguisuelas et al. (28)	Spain	RCT	46.60 (10.30)	46.40 (11.40)	27	27	11/16	10/17	4	2
Arguisuelas et al. (18)	Spain	RCT	47.20 (9.80)	48.60 (10.10)	18	18	6/12	6/12	0	0
Boff et al. (13)	Brazil	RCT	38.10 (7.00)	38.70 (6.80)	36	36	29/7	30/6	0	0
Lee et al. (16)	Korea	RCT	61.01 (7.86)	62.05 (5.79)	15	15	7/8	7/8	0	0
Ozsoy et al. (24)	Turkey	RCT	68.04 (2.97)	68.14 (2.57)	22	23	15/7	17/6	1	2
Yu et al. (23)	Korea	RCT	70.40 (3.20)	69.40 (4.10)	20	20	0/20	0/20	0	0
Branchini. (29)	Italy	RCT	48.00 (12.00)	44.00 (8.20)	11	13	4/7	4/9	8

*MG, Myofascial release group; CG, Control group*.

#### Intervention Characteristics and Outcome Measures

Table 2 summarized the interventions, length, frequency, and duration of interventions, outcomes, and adverse events from the eight RCTs. In terms of intervention comparison between myofascial release group and control group, myofascial release vs. sham myofascial release was used in three studies (18, 22, 28), myofascial release + exercises vs. exercises were used in two studies (16, 24), myofascial release vs. exercises was used in one study (23), Myofascial release + spinal manipulation vs. Spinal manipulation was used in one study (13) and Myofascial release + physiotherapy program vs. physiotherapy program was used in one study (29).

**Table 2:** Intervention characteristics and outcome measures.

**References**	**Intervention length, frequency, and duration**		**Main outcomes and results**	**Adverse event**
	**MG**	**CG**		
Ajimsha et al. (22)	Myofascial release (40 min each; Once every 3 weeks; 8 weeks)	Sham myofascial release	1. Pain (MPQ)^*^; 2. Physical Function (QBPDS)^*^;	10 patients from the MFR group and 1 from control group reported an increase of pain in the first week after initiation of treatment,and this was reported to have subsided within a week without any medications.
Arguisuelas et al. (28)	Myofascial Release (40 min each; Once every 2 weeks; 2 weeks)	Sham myofascial release	1. Pain (MPQ); 2. Physical Function (RMQ)^*^; 3. Mental health (FABQ)^*^;	None
Arguisuelas et al. (18)	Myofascial Release (40 min each; Once every 2 weeks; 2 weeks)	Sham myofascial release	1. Pain (MPQ)^*^; 2. Physical Function (RMQ)^*^;	Not reported
Boff et al. (13)	Myofascial release (6 times in total) + spinal manipulation	Spinal manipulation	1. Pain (VAS); 2. Physical Function (QBPDS); 3. Quality of life (EQ-5-D-3-L); 4. Balance Function (YBT); 5. Pain pressure-thresholds (PPTs);	None
Lee et al. (16)	Dynamic Myofascial Release (15 min each; Once every 2 weeks; 4 weeks) + exercise therapy	Exercise therapy	1. Balance Function (FRT)^*^; 2. Trunk mobility (sagittal plane)^*^; 3. Trunk mobility (coronal plane)^*^;	Not reported
Ozsoy et al. (24)	Myofascial release technique (Once every 3 weeks; 6 weeks) + exercise therapy	Exercise therapy	1. Pain (VAS); 2. Physical Function (ODI); 3. Pain pressure-thresholds (PPTs); 4. Quality of life (WHOQOL-OLD); 5. Trunk mobility (sagittal plane)^*^; 6. Trunk mobility (coronal plane); 7. Mental health (TSK);	Not reported
Yu et al. (23)	Myofascial release (40 min each; Once every 3 weeks; 8 weeks)	Exercise therapy	1. Pain (VAS); 2. Trunk mobility (sagittal plane);	Not reported
Branchini (29)	Myofascial release (45 min each; Twice a week; 4 weeks) + physiotherapy program	Physiotherapy program	Pain (VAS) ^*^; 2. Physical Function (RMQ) ^*^; 3. Quality of life (SF-36) ^*^;	Not reported

*MG, Myofascial release group; CG, Control group; VAS, visual analog scale; MPQ, McGill Pain Questionnaire; QBPDS, Quebec Back Pain Disability Scale; RMQ, Roland Morris Questionnaire; ODI, Oswestry Disability Index; EQ5D3L, EuroQol-5-Dimensions-3-levels; WHOQOL-OLD, World Health Organization Quality of Life Instrument-Older Adults Module; YBT, Y-Balance Test; FRT, Functional Reach Test; FABQ, Fear-Avoidance Beliefs Questionnaire; TSK, Tampa Scale of Kinesiophobia; PPTs, Pain pressure-thresholds; SF-36, The MOS 36-item Short-Form Health Survey*.

^*^ *Denotes sign. post-interventional group differences in favor of myofascial release group*.

Eight included RCTs reported different measurement results, including pain, physical function, quality of life, balance function, pain pressure-threshold, trunk mobility, and mental health. Seven studies assessed pain used MPQ (18, 22, 28) or VAS scores (13, 23, 24, 29), respectively. Six studies assessed physical function used QBPDS (13, 22), RMQ (18, 28, 29), or ODI (24), respectively. Three studies assessed the quality of life used EQ5D3L (13), WHOQOL-OLD (24), or SF-36 (29), respectively. Two studies used YBT (13) or FRT (16) to assess balance function, respectively. Two studies used FABQ (28) or TSK (24) to assess mental health, respectively.

#### Risk of Bias and Quality Assessment

**Figure 2:**
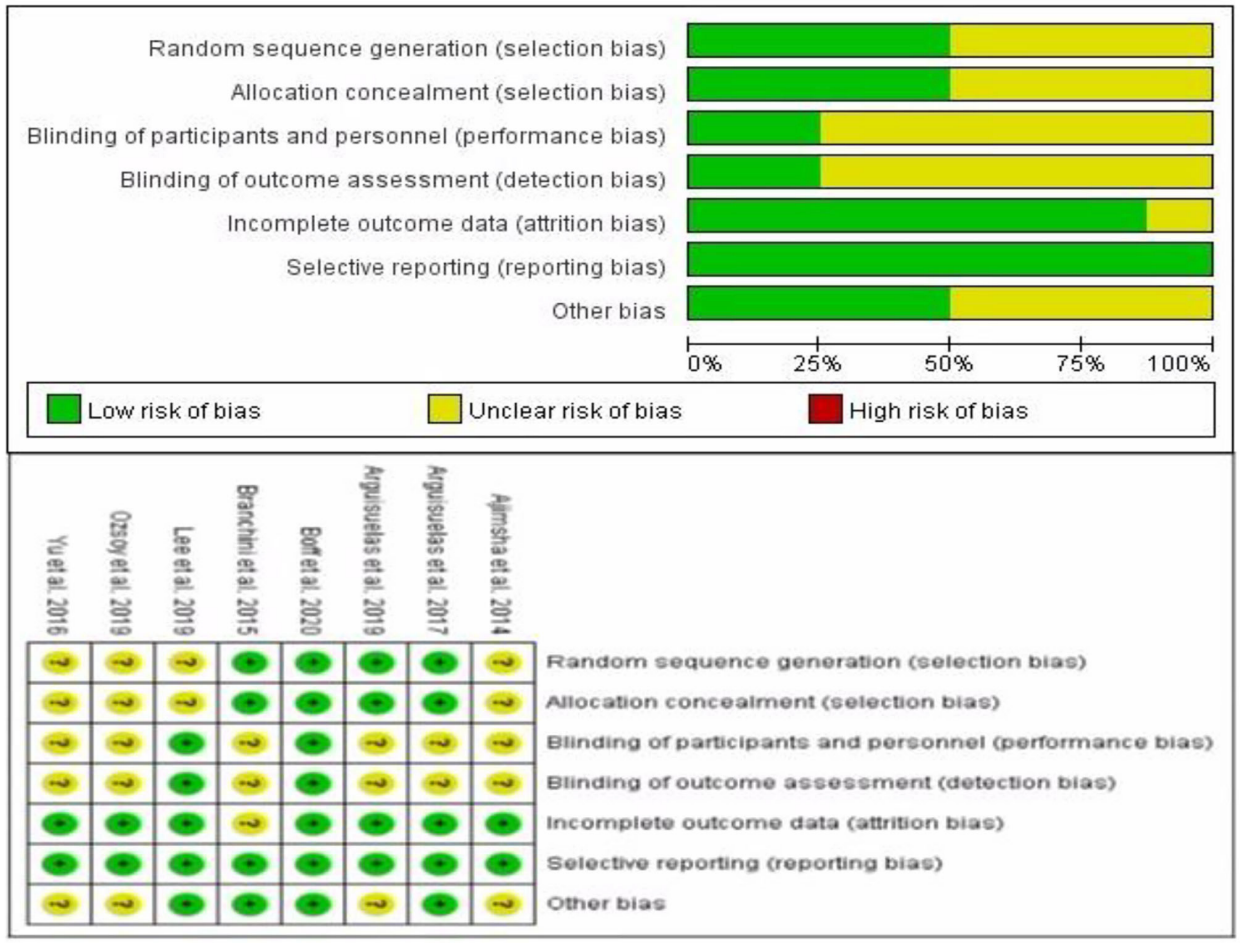
Risk of bias graph.

Figure 2 showed the risk of bias based on the Cochrane HandBook assessment. All studies were described as randomized; four studies described specific randomization methods (13, 18, 28, 29), the other four studies did not describe specific randomization methods (16, 22–24), and the allocation hiding of these four studies was not clear. The performance bias of six RCTs was still unclear (18, 22–24, 28, 29), and the detection bias of these six RCTs were also unclear. Attrition bias in one study was not clear (29). Of the eight RCTs included, only one RCTs had a low risk of bias (13).

#### Quality of Evidence

We used a GRADE system to assess the level of evidence quality for each outcome. The level of evidence quality of pain and physical function was rated as moderate. The level of evidence quality of balance function, WOMAC (total), and trunk mobility were rated as low. Quality of life, pain pressure-threshold, and mental health were rated as very low (Table 3).

**Table 3:** Evidence quality rated using the GRADE approach.

**Outcomes**	**No. of studies**	**Limitations**	**Inconsistency**	**Indirectness**	**Imprecision**	**Publication bias**	**Evidence quality**	
Pain	7	Serious	Not serious	Not serious	Not serious	Undetected	⊕⊕⊕⊖	Moderate
Physical function	6	Serious	Not serious	Not serious	Not serious	Undetected	⊕⊕⊕⊖	Moderate
Quality of life	3	Serious	Serious	Not serious	Serious	Undetected	⊕⊖⊖⊖	Very low
Balance function	2	Serious	Not serious	Not serious	Serious	Undetected	⊕⊕⊖⊖	Low
Pain pressure-threshold	2	Serious	Serious	Not serious	Serious	Undetected	⊕⊖⊖⊖	Very low
Trunk mobility	5	Serious	Not serious	Not serious	Serious	Undetected	⊕⊕⊖⊖	Low
Mental health	2	Serious	Serious	Not serious	Very serious	Undetected	⊕⊖⊖⊖	Very low

### Assessment of Overall Effect Size

#### Pain

**Figure 3:**
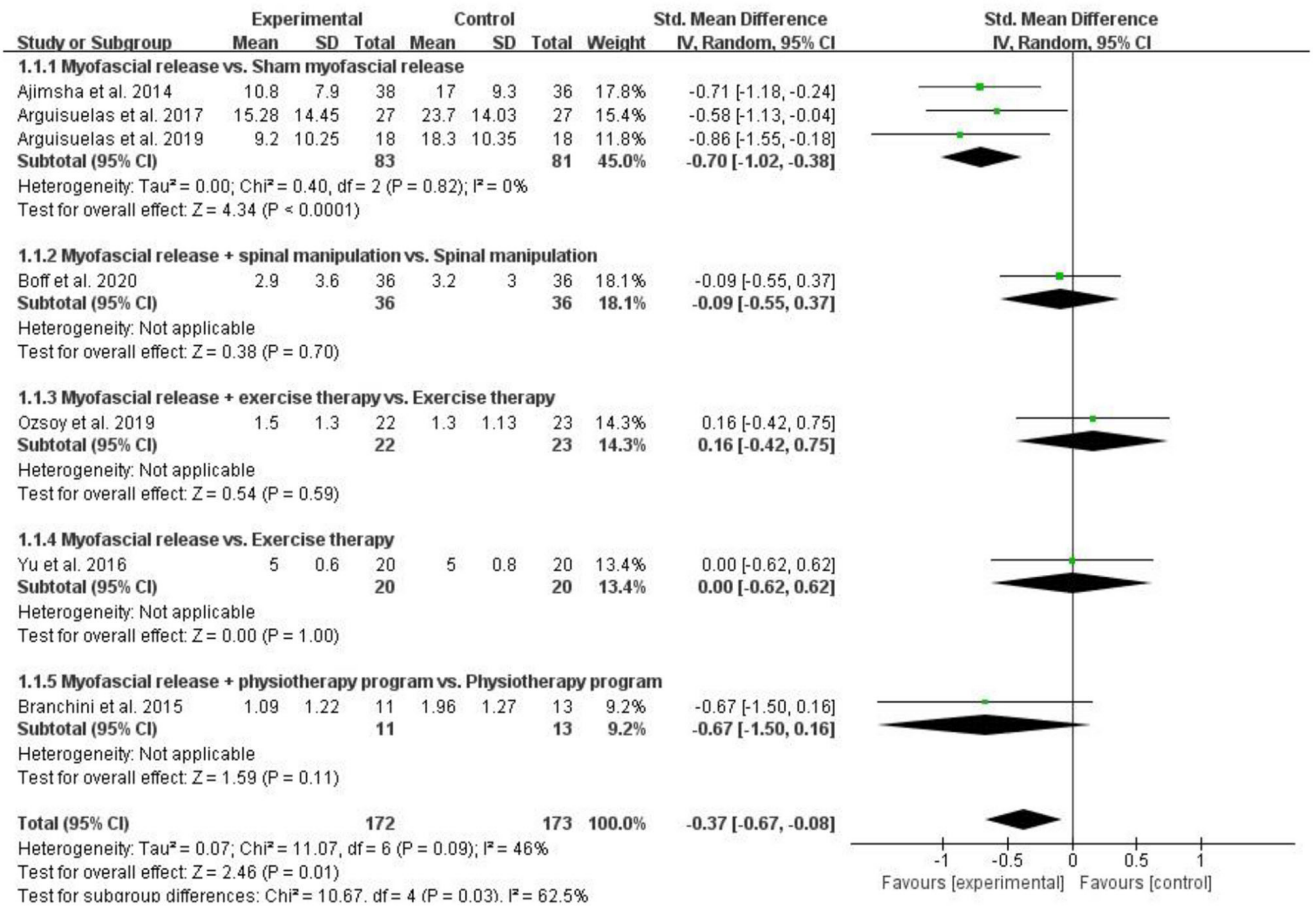
Meta-analysis on pain.

Seven RCTs assessed pain and included 345 patients with CLBP. The pain was assessed by VAS (13, 23, 24, 29) or MPQ (18, 22, 28) in 7 RCTs, respectively. The higher the score on these scales, the more severe the pain. Pooled results showed a significant improvement in pain in the myofascial release group compared to the control group [SMD = −0.37, 95% CI (−0.67, −0.08), *I*^2^ = 46%, *P* = 0.01]. When the myofascial release was compared with the sham myofascial release, the subgroup analysis showed significant improvement in pain in the Myofascial release group [SMD = −0.70, 95% CI (−1.02, −0.38), *I*^2^ = 0%, *P* < 0.0001]. When myofascial release + spinal manipulation was compared with spinal manipulation, subgroup analysis showed no significant improvement in pain in the myofascial release group [SMD = −0.09, 95% CI (−0.55, 0.37), *P* = 0.70]. When myofascial release + exercise therapy was compared with exercise therapy, subgroup analysis showed no improvement in pain in the myofascial release group [SMD = 0.16, 95% CI (−0.42, 0.75), *P* = 0.59]. Similarly, when the myofascial release was compared with exercise therapy, subgroup analysis showed no improvement in pain in the myofascial release group [SMD = 0.00, 95% CI (−0.62, 0.62), *P* = 0.11]. In addition, when the myofascial release + physiotherapy program was compared with the physiotherapy program, subgroup analysis showed no significant improvement in pain in the myofascial release group [SMD = −0.37, 95% CI (−0.67, −0.08), *P* = 0.70] (Figure 3).

#### Physical Function

**Figure 4:**
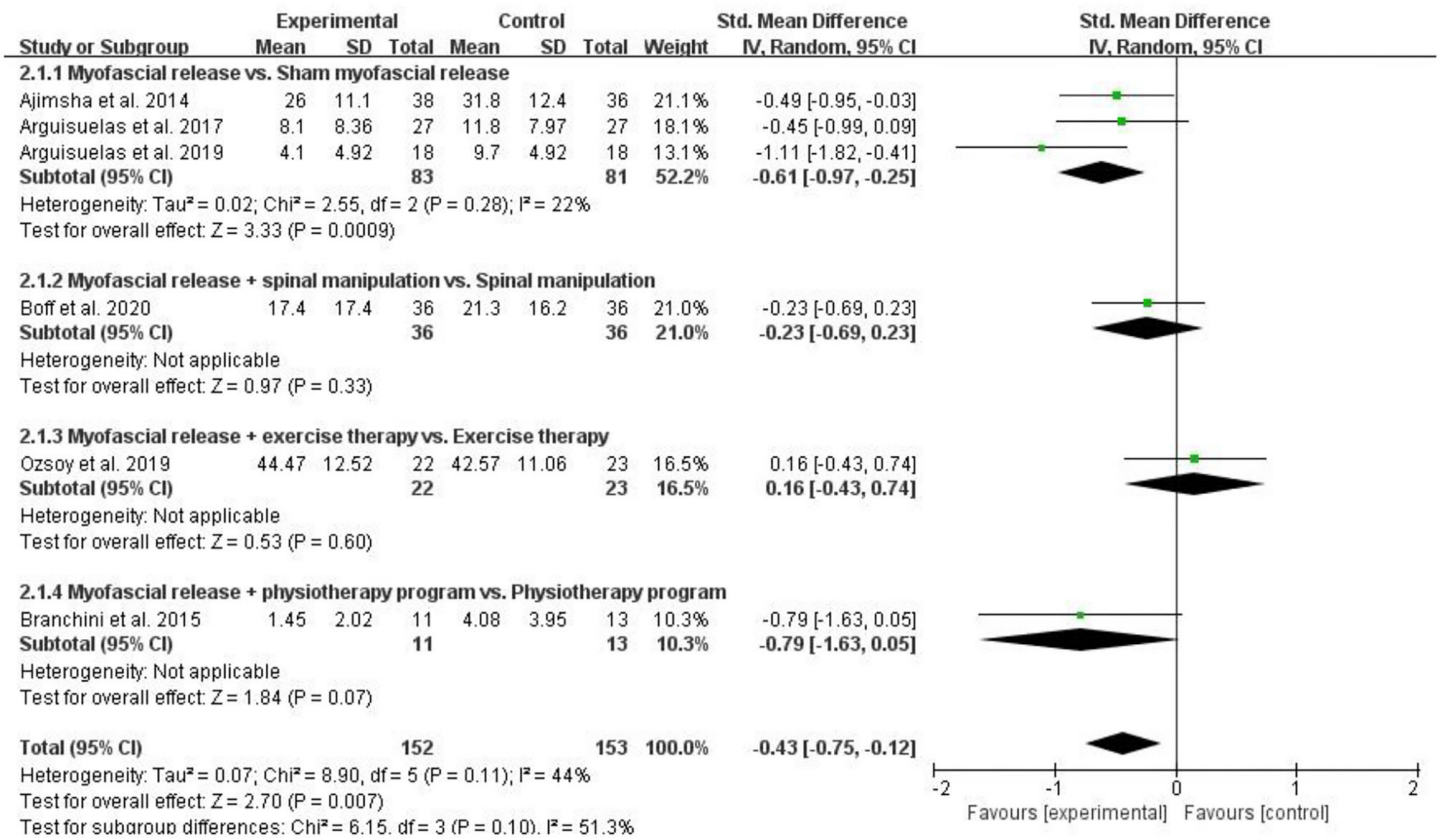
Meta-analysis on physical function.

Six RCTs assessed physical function and included 305 patients with CLBP. Six RCTs assessed physical function used QBPDS (13, 22), RMQ (18, 28, 29), or ODI (24), respectively. The higher the score on these scales, the worse the physical function. Pooled results showed a significant improvement in physical function in the myofascial release group compared to the control group [SMD = −0.43, 95% CI (−0.75, −0.12), *I*^2^ = 44%, *P* = 0.007]. When the myofascial release was compared with sham myofascial release, subgroup analysis showed significant improvement in physical function in the myofascial release group [SMD = −0.61, 95% CI (−0.97, −0.25), *I*^2^ = 22%, *P* = 0.0009]. However, when myofascial release + spinal manipulation was compared with spinal manipulation, subgroup analysis showed no significant improvement in the physical function of the myofascial release group [SMD = −0.23, 95% CI (−0.69, 0.23), *P* = 0.33]. When myofascial release + exercise therapy was compared with exercise therapy, subgroup analysis showed no improvement in the physical function of the myofascial release group [SMD = 0.16, 95% CI (−0.43, 0.74), *P* = 0.60]. In addition, when the myofascial release + physiotherapy program was compared with the physiotherapy program, subgroup analysis showed that the myofascial release group does not significantly improve its physical function [SMD = −0.79, 95% CI (−1.63, 0.05), *P* = 0.70] (Figure 4).

#### Quality of Life

**Figure 5:**
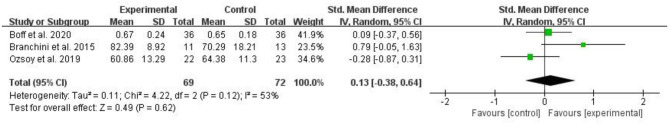
Meta-analysis on quality of life.

Three RCTs assessed the quality of life and included 141 patients with CLBP. Three RCTs assessed the quality of life used EQ5D3L (13), WHOQOL-OLD (24), or SF-36 (29), respectively. The higher the score on these scales, the better the quality of life. The pooled results showed no significant improvement in quality of life in the myofascial release group compared to the control group [SMD = 0.13, 95% CI (−0.38, 0.64), *I*^2^ = 53%, *P* = 0.62] (Figure 5).

#### Balance Function

**Figure 6:**
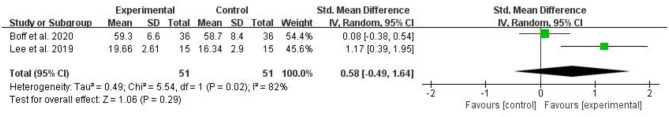
Meta-analysis on balance function.

Two RCTs evaluated balance function and included 102 patients with CLBP. Two RCTs assessed the balance function used YBT (13) or FRT (16). The higher the value of these measurements, the better the balance function. The pooled results showed no significant improvement in quality of life in the myofascial release group compared to the control group [SMD = 0.58, 95% CI (−0.49, 1.64), *I*^2^ = 82%, *P* = 0.29] (Figure 6).

#### Pain Pressure-Threshold

**Figure 7:**
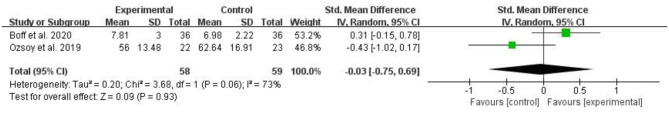
Meta-analysis on pain pressure-threshold.

Two RCTs assessed the pain pressure-threshold and included 117 patients with CLBP. The pooled results showed that the pain pressure-threshold of the myofascial release group was not significantly increased compared with the control group [SMD = 0.03, 95% CI (−0.75, 0.69), *I*^2^ = 73%, *P* = 0.93] (Figure 7).

#### Trunk Mobility

**Figure 8:**
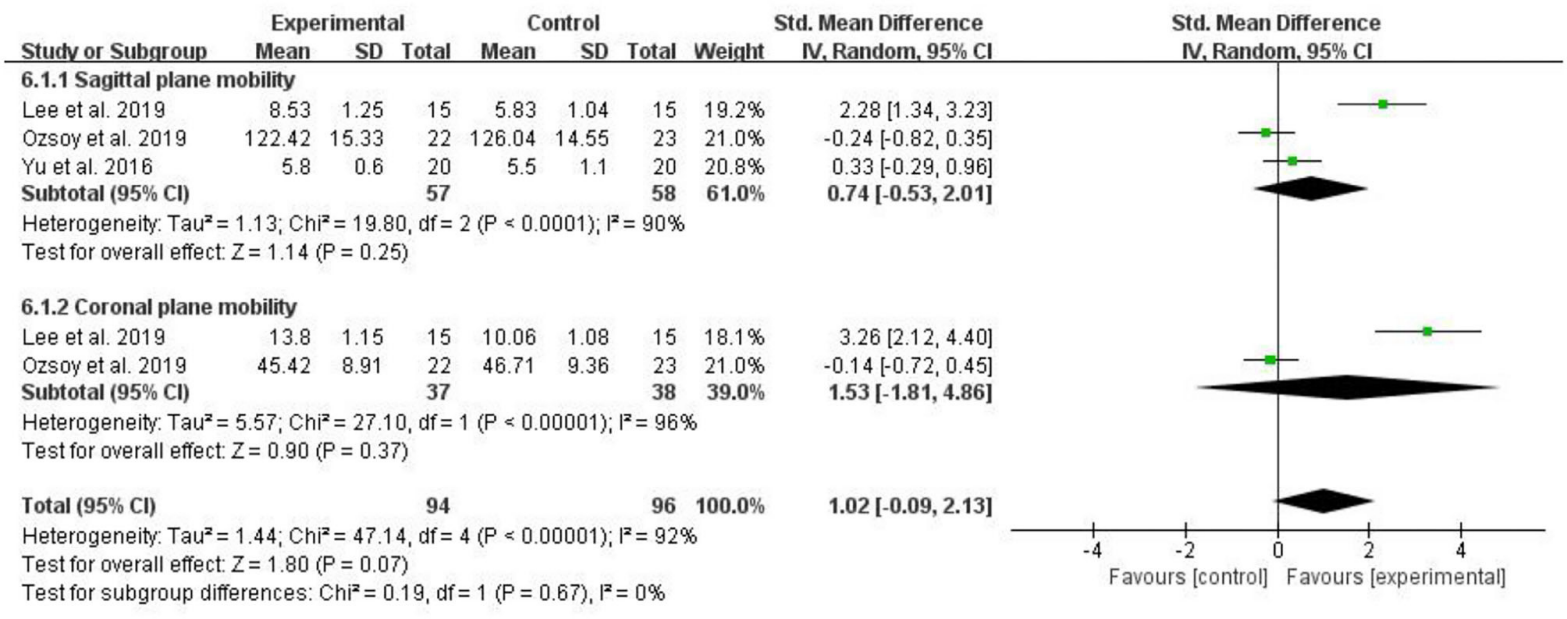
Meta-analysis on trunk mobility.

Five RCTs evaluated trunk mobility and included 190 patients with CLBP. The larger the value of these measurements, the better the patient's trunk mobility. The pooled results showed that compared with the control group, trunk mobility in the myofascial release group did not significantly improve [SMD = 1.02, 95% CI (−0.09, 2.13), *I*^2^ = 92%, *P* = 0.07]. Subgroup analysis showed that there was no significant difference in trunk mobility (Sagittal plane mobility) between the myofascial release group and the control group [SMD = 0.74, 95% CI (−0.53, 2.01), *I*^2^ = 90%, *P* = 0.25]. Similarly, subgroup analysis showed that trunk mobility (Coronal plane mobility) between the myofascial release group and the control group was not significantly different [SMD = 1.53, 95% CI (−1.81, 4.86), *I*^2^ = 96%, *P* = 0.37] (Figure 8).

#### Mental Health

**Figure 9:**
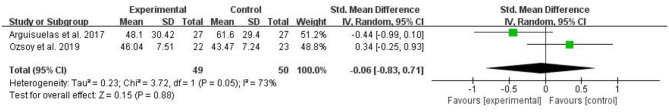
Meta-analysis on mental health.

Two RCTs evaluated mental health and included a total of 99 patients with CLBP. Two RCTs assessed mental health using FABQ (28) or TSK (24). The higher the value of these measures, the worse the mental health. The pooled results showed no significant improvement in mental health in the myofascial release group compared to the control group [SMD = −0.06, 95% CI (−0.83, 0.71), *I*^2^ = 73%, *P* = 0.88] (Figure 9).

### Publication Bias

**Figure 10:**
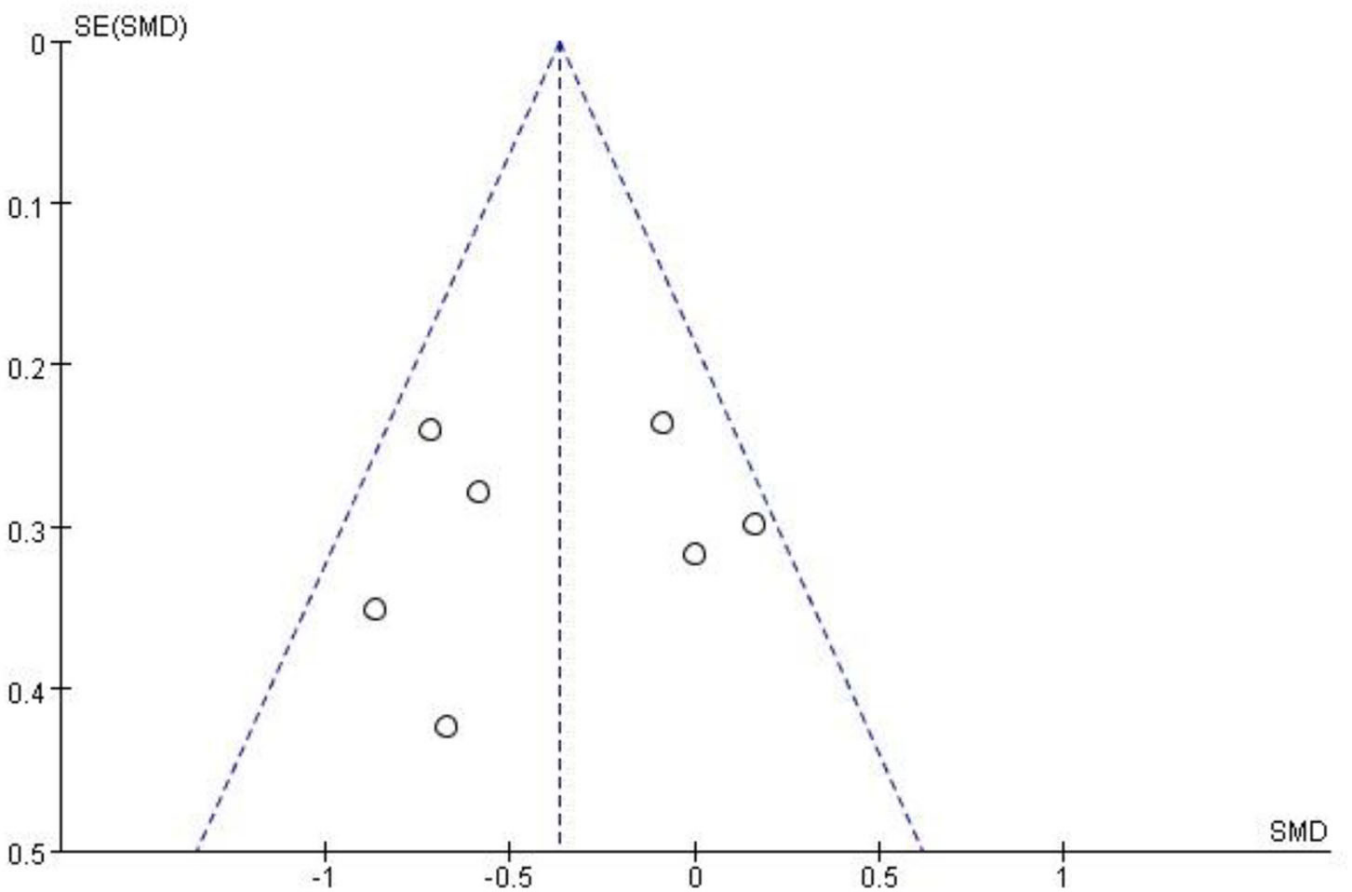
Funnel plot.

When more than ten studies were included in the meta-analysis, the publication bias of these studies should be evaluated. None of the studies we included had more than 10 (30). However, we used funnel plots to assess publication bias for pain in the largest number of included studies. The funnel plot was symmetric, indicating that there was no publication bias (Figure 10).

#### Adverse Events

Of the eight included RCTs, one RCTs reported adverse events (22), two RCTs reported no adverse events (13, 28), and the other five RCTs did not report adverse events (16, 18, 23, 24, 29).

## Discussion

### Summary of Evidence

We conducted this review to evaluate the scientific evidence for the benefits of myofascial release interventions in patients with CLBP compared to non-myofascial release interventions. The major outcomes of the assessment include pain, physical function, quality of life, balance function, pain pressure-threshold, and minor outcomes include trunk mobility and mental health. The meta-analysis results indicate that myofascial release may help improve the pain and physical function of patients with CLBP. However, when we conducted a subgroup analysis of different interventions, we found that different interventions would produce different results. Meanwhile, the meta-analysis results also showed that myofascial release had no significant effect on the quality of life, balance function, pain pressure-threshold, trunk mobility, and mental health in patients with CLBP.

### Comparison to Prior Studies

To the best of our knowledge, this is the first systematic review and meta-analysis to evaluate the effectiveness of myofascial release in CLBP. In recent years, myofascial release has become more widely used in clinical practice, especially in treating CLBP. At the same time, more and more related RCTs have been published, but no systematic reviews and meta-analyses have been carried out. In this study, we included eight RCTs, of which four RCTs concluded that myofascial release is effective in treating CLBP (16, 22, 28, 29), and three RCTs concluded that myofascial release is effective for some indicators of CLBP (18, 23, 24), and another randomized controlled trial concluded that myofascial release has no significant effect on the treatment of CLBP (13). Overall, the meta-analysis results showed that myofascial release improved pain and physical function in patients with CLBP but had no significant effect on the quality of life, balance function, pain pressure-threshold, trunk mobility, and mental health. Due to the small number and low quality of the included literature, these conclusions are only preliminary, and more high-quality clinical studies are needed in the future.

### Is Myofascial Release an Effective Treatment for Chronic Low Back Pain?

Chronic low back pain is one of the main causes of pain (31, 32), dysfunction, and disability, and it puts enormous pressure on the society, medical, and welfare system (33–35). The diagnosis and treatment of CLBP is a hot topic. At present, there are a series of clinical interventions to treat CLBP, but they often lack clinical effectiveness (36). In this case, some researchers believe that myofascial release may be a suitable method for treating CLBP (17, 18). Myofascial release is described as a general term for various manual treatment techniques that exert pressure on muscles and myofascial, which aims to relieve pain by restoring the function of damaged soft tissues (17, 37). The origin of myofascial release can be traced back to the 1940s, but the term myofascial release was not proposed until 1981 (38). Myofascial tissue may be the source of pain in some musculoskeletal diseases, such as plantar fasciitis and CLBP (16). The theory of the therapeutic effect of myofascial release is based on the special role of fascia. The theory holds that myofascial is the main factor determining musculoskeletal function and plays a vital role in the dynamic characteristics of the human body (39). Fascial tissue hardening or increased tension and decreased sliding ability may be the cause of tension in other parts of the body, which in turn leads to increased pain and limited function (39–42). Myofascial release therapy uses stretch-restricted myofascial so that the length and performance of the myofascial membrane return to normal (25, 39, 40). Meanwhile, myofascial release can reduce the pressure on pain-sensitive structures such as nerves and blood vessels by improving the length and health of restricted connective tissues (22). Studies have found that myofascial release combined with conventional treatment can significantly improve the body's pain and tenderness (37, 43). Although the specific mechanism of myofascial release is not yet clear, studies have found that myofascial release stimulates receptors distributed in the myofascial membrane, leading to neuromuscular changes (18). In addition, *in vitro* studies have found that myofascial release can also reduce the production of inflammatory cytokines (44).

The central nervous system regulation of pain may be altered due to the occurrence of CLBP (45). It has been reported that most patients with CLBP do not have spine-related pathological changes but have chronic musculoskeletal dysfunction and that treatment of these musculoskeletal disorders can effectively relieve the pain of patients (46). Pain level is closely related to body function (47). By improving pain, body function recovery can be promoted. At the same time, myofascial release may improve the patient's physical function by improving the patient's exercise status and trunk mobility (48). Myofascial release can improve pain, improve body flexibility, and thus increase trunk mobility (23). Previous studies have shown that the pain threshold of patients with CLBP is lower than that of healthy people, and the reduction of pain threshold is related to the decrease of the intensity of CLBP and the reduction of physical function (49–51). In addition, changes in pain pressure-threshold and balance function as well as weakening of strength will affect the movement control ability of patients with CLBP and may lead to the recurrence of CLBP (52–54), and improving trunk exercise ability can further improve balance function (16). An increase in pain leads to a decrease in balance function (23). The maintenance of balance function requires the integration of sensory information input, central nervous system processing, and neuromuscular activity (53, 55). The decreased stability of the body in patients with CLBP may be due to impaired proprioception (54), and some manual treatments can stimulate the proprioceptors to have a positive effect on posture control and body stability (55, 56). Myofascial release promotes the increase of trunk mobility through biomechanical effects and improves the patient's balance function through the overall adjustment of the nervous system (57, 58). Myofascial release can relax the tense tissues that cause pain, thereby inducing the imbalance of the body to a balanced and stable state (23). In addition, the degree of pain and disability in patients with CLBP may have a negative impact on the patient's quality of life (59), and myofascial release may improve the patient's quality of life by improving pain and disability. At the same time, more and more researchers have found that psychosocial factors play a vital role in CLBP (60). Some researchers have found that even in pain control, psychological factors still affect the lumbar spine mobility and cause abnormal muscle activity in patients with CLBP (61, 62). This meta-analysis showed that myofascial release could improve pain and physical function in patients with CLBP. We also analyzed the effects of myofascial release on balance function, pain pressure-threshold, trunk mobility, mental health, and quality of life in patients with CLBP. However, we did not find a significant effect in this meta-analysis, which may be due to the small number of included studies and possible publication bias.

### Limitations and Quality of Evidence

Although we included the literature strictly according to the criteria, this study had several limitations. First, the included studies had some risk of bias in terms of randomization methods, allocation concealment, implementation bias, and detection bias, which reduced the quality of the literature. Second, the small number of included literature may affect the comparison of differences between groups. Although a small number of studies can be used for meta-analysis, the conclusion should be regarded as preliminary (27). Third, most of the included studies did not have long-term follow-up. Follow-up was conducted in some studies, but the duration of follow-up varied greatly between different studies, so we could not conduct subgroup analysis according to different follow-up times. Therefore, we extracted data from the longest follow-up after the end of the intervention to assess long-term outcomes. Fourth, different forms of myofascial release were used in this study. There may be differences in the efficacy of different forms of myofascial release techniques, which may have a potential impact on our results, and this may also explain the high heterogeneity. Fifth, although the included studies reported disease duration, they were not grouped by disease duration. Therefore, we could not evaluate the efficacy of myofascial release in patients with different severity of CLBP.

### Implications for Further Research

Although there are some difficulties in conducting RCTs, future studies should adopt more rigorous designs. First, future RCTs should strictly follow the CONSORT guidelines to reduce the risk of bias (63). Second, when conducting clinical studies on myofascial release, the duration and frequency of follow-up should be extended to evaluate the short- and long-term effects of myofascial release for CLBP. Third, future RCTs should strictly limit interventions and reduce the use of combination interventions. Myofascial release as an intervention in the intervention group should be compared directly with other interventions. Fourth, when conducting RCTs, patients with CLBP with different levels of severity should be grouped, and different levels of CLBP may have different outcomes. Fifth, it should be registered in the clinical trial center before the start of the research. It is recommended that researchers publish the complete research plan to reduce selective reports (64). Finally, due to the methodological limitations of the included literature, more high-quality RCTs should be conducted in the future to verify the current conclusions.

## Conclusion

In this study, we systematically reviewed and quantified the efficacy of myofascial release in treating CLBP. The meta-analysis results showed that myofascial release significantly improved pain and physical function in patients with CLBP but had no significant effects on balance function, pain pressure-threshold, trunk mobility, mental health, and quality of life. Due to the low quality and a small number of included literature, more and more rigorously designed RCTs should be included in the future to verify these conclusions.

## Data Availability Statement

The original contributions presented in the study are included in the article/Supplementary Material, further inquiries can be directed to the corresponding author/s.

## Author Contributions

ZW, YZ, GC, and XX designed the study. ZW, YW, XY, and ZC conducted literature search and screening, extracted data from the literature, and statistical analysis. RZ, ZY, and JH checked the extracted data. ZW, RZ, ZY, and JH wrote the first draft. YZ, GC, and XX corrected the manuscript and supervised the conduct of the study. All authors have read and approved the final submitted version.

## Conflict of Interest

The authors declare that the research was conducted in the absence of any commercial or financial relationships that could be construed as a potential conflict of interest.

## Publisher's Note

All claims expressed in this article are solely those of the authors and do not necessarily represent those of their affiliated organizations, or those of the publisher, the editors and the reviewers. Any product that may be evaluated in this article, or claim that may be made by its manufacturer, is not guaranteed or endorsed by the publisher.
