# Supplementary material for: Myofascial Release for Chronic Low Back Pain: A Systematic Review and Meta-Analysis
Source: Front Med (Lausanne). 2021 Jul 28;8:697986. doi: 10.3389/fmed.2021.697986 (PMC8355621; doi:10.3389/fmed.2021.697986)
Supplement: Supplementary file 1 [file Data_Sheet_1.docx]

**Myofascial Release For Chronic Low Back Pain: A Systematic Review and Meta-Analysis**

**Supplementary Appendix -** **Search algorithm**

**PubMed:**

*#1 ((((((((((((((((((((((((((((((((((((Low Back Pain[MeSH Terms]) OR (Chronic low back pain[MeSH Terms])) OR (Nonspecific low back pain[MeSH Terms])) OR (Back Pain[MeSH Terms])) OR (Low Back Pain[Title/Abstract])) OR (Chronic low back pain[Title/Abstract])) OR (Nonspecific low back pain[Title/Abstract])) OR (Back Pain[Title/Abstract])) OR (Back Pain, Low[Title/Abstract])) OR (Back Pains, Low[Title/Abstract])) OR (Low Back Pains[Title/Abstract])) OR (Pain, Low Back[Title/Abstract])) OR (Pains, Low Back[Title/Abstract])) OR (Lumbago[Title/Abstract])) OR (Lower Back Pain[Title/Abstract])) OR (Back Pain, Lower[Title/Abstract])) OR (Back Pains, Lower[Title/Abstract])) OR (Lower Back Pains[Title/Abstract])) OR (Pain, Lower Back[Title/Abstract])) OR (Pains, Lower Back[Title/Abstract])) OR (Low Back Ache[Title/Abstract])) OR (Ache, Low Back[Title/Abstract])) OR (Aches, Low Back[Title/Abstract])) OR (Back Ache, Low[Title/Abstract])) OR (Back Aches, Low[Title/Abstract])) OR (Low Back Aches[Title/Abstract])) OR (Low Backache[Title/Abstract])) OR (Backache, Low[Title/Abstract])) OR (Backaches, Low[Title/Abstract])) OR (Low Backaches[Title/Abstract])) OR (Low Back Pain, Postural[Title/Abstract])) OR (Postural Low Back Pain[Title/Abstract])) OR (Low Back Pain, Posterior Compartment[Title/Abstract])) OR (Low Back Pain, Recurrent[Title/Abstract])) OR (Recurrent Low Back Pain[Title/Abstract])) OR (Low Back Pain, Mechanical[Title/Abstract])) OR (Mechanical Low Back Pain[Title/Abstract])*

*67,252*

*#2 (Myofascial release[MeSH Terms]) OR (Myofascial release[Title/Abstract]) 345*

*#3 #1 AND #2 32*

*#4 ((((((((Randomized Controlled Trial[Publication Type]) OR (Clinical Trial[Publication Type])) OR (Randomly[Title/Abstract])) OR (Randomised[Title/Abstract])) OR (random[Title/Abstract])) OR (randomization[Title/Abstract])) OR (Trial[Title/Abstract])) OR (Control[Title/Abstract])) OR (Controlled[Title/Abstract]) 4,422,131*

*#5 #3 AND #4 14*

**EMBASE:**

*#1 'low back pain'/exp 60,020*

*#2 'chronic low back pain'/exp 60,020*

*#3 'backache'/exp 116,137*

*#4 'nonspecific low back pain'/exp 24*

*#5 'low back pain':ab,ti 37,697*

*#6 'chronic low back pain':ab,ti 9,034*

*#7 'nonspecific low back pain':ab,ti 793*

*#8 'back pain':ab,ti 69,388*

*#9 'lumbago':ab,ti 1,879*

*#10 'low back ache':ab,ti 108*

*#11 'back pains':ab,ti 292*

*#12 'lower back pains':ab,ti 17*

*#13 'low backache':ab,ti 307*

*#14 #1 OR #2 OR #3 OR #4 OR #5 OR #6 OR #7 OR #8 OR #9 OR #10 OR #11 OR #12 OR #13 128,506*

*#15 'myofascial release'/exp 50*

*#16 'myofascial release technique'/exp 10*

*#17 'myofascial release':ab,ti 413*

*#18 'myofascial release technique':ab,ti 26*

*#19 #15 OR #16 OR #17 OR #18 430*

*#20 'randomized controlled trial'/exp 643,171*

*#21 'randomized controlled trial':ti 55,313*

*#22 #20 OR #21 649,137*

*#23 #14 AND #19 AND #22 10*

**Cochrane Library:**

*#1 MeSH descriptor: [Low Back Pain] explode all trees 3,870*

*#2 MeSH descriptor: [Back Pain] explode all trees 5,008*

*#3 ("low back pain"):ti,ab,kw 10,426*

*#4 ("chronic low back pain"):ti,ab,kw 3,743*

*#5 (Nonspecific low back pain):ti,ab,kw 1,554*

*#6 (Non-specific low back pain):ti,ab,kw 1,072*

*#7 ("back-pain"):ti,ab,kw 13,806*

*#8 ("back pain"):ti,ab,kw 13,806*

*#9 ("lumbago"):ti,ab,kw 235*

*#10 (Low Back Ache):ti,ab,kw 71*

*#11 (Lower Back Pains):ti,ab,kw 3176*

*#12 (Low Backache):ti,ab,kw 860*

*#13 (Low Backaches):ti,ab,kw 859*

*#14 (Postural Low Back Pain):ti,ab,kw 674*

*#15 (Recurrent Low Back Pain):ti,ab,kw 465*

*#16 (Mechanical Low Back Pain):ti,ab,kw 995*

*#17 #1 OR #2 OR #3 OR #4 OR #5 OR #6 OR #7 OR #8 OR #9 OR #10 OR #11 OR #12 OR #13 OR #14 OR #15 OR #16 15,273*

*#18 (myofascial release):ti,ab,kw 415*

*#19 (myofascial release technique):ti,ab,kw 191*

*#20 #18 OR #19 415*

*#21 #17 AND #20 62*

**Web of Science:**

*#1 TS=Low Back Pain 72,904*

*#2 TS=Chronic low back pain 21,508*

*#3 TS=Nonspecific low back pain 1,878*

*#4 TS=Back Pain 111,769*

*#5 TI=Low Back Pain 18,957*

*#6 TI=Chronic low back pain 5,838*

*#7 TI=Nonspecific low back pain 570*

*#8 TI=Non-specific low back pain 680*

*#9 TI=Back Pain 25,861*

*#10 TI=Lumbago 619*

*#11 TI=Low Back Ache 22*

*#12 TI=Lower Back Pain 18,961*

*#13 TI=Low Backache 172*

*#14 TI=Postural Low Back Pain 208*

*#15 TI=Recurrent Low Back Pain 160*

*#16 TI=Mechanical Low Back Pain 202*

*#17 #1 OR #2 OR #3 OR #4 OR #5 OR #6 OR #7 OR #8 OR #9 OR #10 OR #11 OR #12 OR #13 OR #14 OR #15 OR #16 111,860*

*#18 TS=myofascial release 828*

*#19 TI=myofascial release 214*

*#20 AB=myofascial release 616*

*#21 AK=myofascial release 168*

*#22 TS=myofascial release technique 369*

*#23 TI=myofascial release technique  31*

*#24 AB=myofascial release technique  278*

*#25 AK=myofascial release technique 16*

*#26 #18 OR #19 OR #20 OR #21 OR #22 OR #23 OR #24 OR #25 828*

*#27 TS=Randomized Controlled Trial  596,745*

*#28 TI=Randomized Controlled Trial  128,138*

*#29 AB=Randomly 469,487*

*#30 AB=Randomised 790,416*

*#31 AB=random 784,143*

*#32 AB=randomization 48,962*

*#33 #27 OR #28 OR #29 OR #30 OR #31 OR #32 2,007,012*

*#34 #17 AND #26 AND #33 57*
